# “Universal health coverage and priority diseases diagnostics: a case study of Essential Package of Health Services from Pakistan”

**DOI:** 10.1016/j.lansea.2026.100719

**Published:** 2026-01-19

**Authors:** Mohammad Zeeshan, Sadia Shakoor, Amna Rehana Siddiqui, Rumina Hasan

**Affiliations:** a Department of Pathology and Laboratory Medicine Aga Khan University Hospital, Stadium Road, Karachi, Pakistan; b APPNA Institute of Public Health Jinnah Sindh Medical University Karachi, Pakistan

Laboratory diagnostic services are vital for disease management and for developing public health strategies. Nevertheless, almost half of the global population continues to face challenges in accessing diagnostic services, limiting efforts toward universal health coverage.^1^ Pakistan has committed to providing universal health coverage (UHC-ACT 2024), thereby ensuring access to quality-assured diagnostic services in the country.^2^

UHC-Service Coverage Index (SCI), a key metric for assessing essential health services has improved globally including in Pakistan. Pakistan's score of 45 (on a scale from 0 to 100) is among the lowest when compared to its neighboring countries.^3^

Sindh province health ministry launched Essential Packages for Health Services (EPHS-S) to strengthen its health system and achieve Universal Health Coverage (UHC). They identified key interventions by comparing existing services with Disease Control Priorities (DCP3) recommendations and the national EPHS framework, also incorporating relevant laboratory tests.^4^ Despite aiming for improved healthcare, the current EPHS-S test menu for primary healthcare (PHC) clinics and first-level hospitals (FLH) with labs in Pakistan falls short of addressing provincial health priorities.

EPHS-S 2021 in vitro diagnostic (IVD) tests were compared against the WHO Essential Diagnostic List (WHO-EDL) 2022, a reference for Universal Health Coverage (UHC). We excluded WHO-EDL tests for a) low-burden diseases in Sindh (e.g., histoplasmosis, trypanosomiasis, visceral leishmaniasis, aspergillosis, pneumocystis, Zika) and high-complexity tests unsuitable for first-level hospital (FLH) labs (e.g., nucleic acid amplification, mutation detection and flow cytometry). For facilities without a lab, 35 tests were included and 8 excluded from analysis. For those with a lab, 114 tests were included and 48 excluded. A comparison of tests between EPHS and WHO-EDL is shown in Fig. 1. PHC clinics EPHS-S included only 4 of 23 relevant WHO-EDL tests, with 5 EPHS-S tests being inadequately defined for UHC. FLHs with labs had 26 of 59 WHO-EDL tests in EPHS-S, and many EPHS-S tests lacked sufficient detail for mapping to the WHO-EDL.

**Fig. 1:**
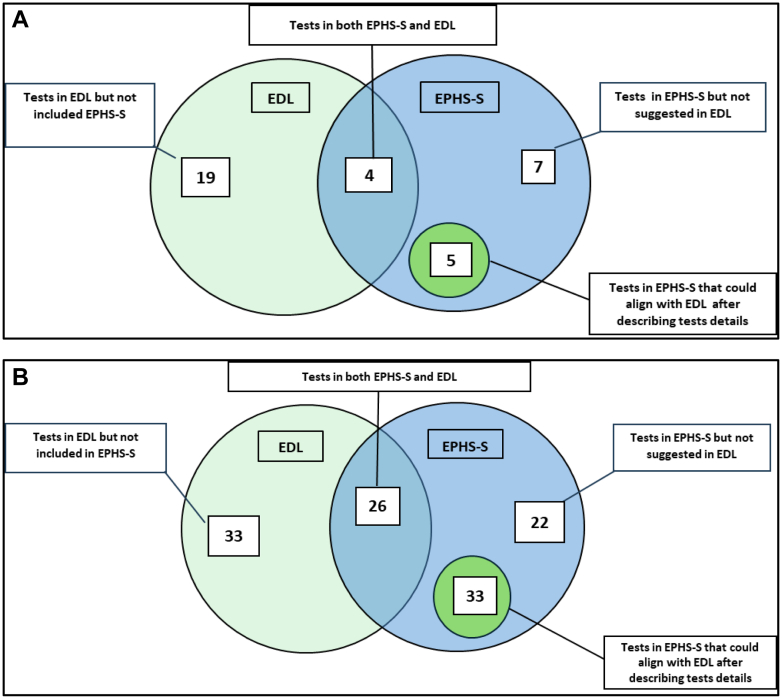
Comparison of tests suggested in EPHS and EDL—**A**: For PHC clinics. **B:** For FLH with a laboratory.

Diagnostic tests not listed in EPHS-S but recommended in WHO-EDL are listed in Supplementary Table S1.

Our analysis shows major gaps in EPHS-S: tests listed fail to address priority high-burden health conditions for Pakistan based on the shifts in Pakistan's Disability-Adjusted Life Years (DALYs) over the past 30 years.

Three major disease areas remain unaddressed in EPHS-S where bridging the in-vitro testing gaps will substantially improve the program's capacity to diagnose and manage these diseases, thereby advancing the goal of UHC.^5^

An epidemic of diabetes has emerged in Pakistan, as indicated by a 274% change in its associated DALYs during the past three decades.^5^ Health systems require strategies for early detection and consistent monitoring. Availability of glycosylated hemoglobin (HbA1C) at health facilities without laboratories through handheld analyzers is expected to increase population access to an initial diagnosis of diabetes, facilitating early management and cost-saving through prevention of several diabetes-potentiated clinical outcomes, as evidenced by cost-effective improvements in other countries.^6^

A 212% increase in CKD-related DALYs highlights the substantial burden contributed by diabetes, hypertension, and stone diseases across Pakistan.^7^ A point-of-care CKD screening strategy that includes urine dipsticks, as suggested by an Indian research group, could effectively identify patients for confirmatory kidney function tests. This would allow for prompt pharmacological interventions to slow ESRD progression in both diabetic and non-diabetic population.^8^

EDL lists urinary ketones and albumin as standalone dipstick tests, whereas EPHS-S includes them within a generalized urine chemistry category without explicit specification. This strategy may enhance healthcare efficiency by reducing the number of referrals to secondary care for CKD diagnosis and the associated costs of ESRD management.

The Sindh health strategy (2012–2020) prioritized early detection and management of hypertension, diabetes, mental health disorders, and breast/cervical cancers.^9^ However, crucial cancer diagnostic services, particularly screening, are lacking in EPHS-S. The addition of basic imaging for screening, appropriate for PHC and FLH, could be a transformative addition to the EPHS in Pakistan. Population-level expansion of cancer screening programs has demonstrated a decrease in caner associated mortality.^10^ Pakistan has a high burden of breast and oral cancer (DALYs—243%); targeting these most prevalent cancers for an early diagnosis and facilitated referral schemes through EPHS will provide an opportunity for cost-saving and improving population health and access to cancer prevention and management.^11^

The effective alignment of the EPHS-S with the EDL will lead to optimize triage to higher level of care facilities.

Improving access to essential diagnostic services for priority diseases at all healthcare levels is crucial for achieving universal health coverage, promoting population health, and addressing health emergencies, aligning with WHO's 13th General Program of Work. India's development of a National Essential Diagnostic List (NEDL) serves as a model.^12^ This case study can guide Pakistan and other regional countries in developing their own NEDLs, tailored to their health priorities. Therefore, we urgently recommend reforming EPHS to include diagnostic tests and strategies for high-burden non-communicable diseases such as diabetes, chronic kidney disease, and cancers.

## Contributors

**MZ**: Conceptualization, literature review, data curation, methodology, formal analysis, manuscript writing, review and final approval.

**SS**: Conceptualization, methodology, critical review and final approval.

**ARS**: Supervision, critical review, editing and final approval.

**RH**: Supervision, conceptualization, methodology, critical review and final approval.

## Declaration of interests

The authors have no conflict of interest to disclose with regard to this article. Views expressed in the paper represent authors' own views and not represent their country or organizations.
