# Supplementary material for: “Universal health coverage and priority diseases diagnostics: a case study of Essential Package of Health Services from Pakistan”
Source: Lancet Reg Health Southeast Asia. 2026 Jan 19;45:100719. doi: 10.1016/j.lansea.2026.100719 (PMC12856640; doi:10.1016/j.lansea.2026.100719)
Supplement: Supplementary Table S1 [file mmc1.docx]

| **General t** | **Facilities without laboratories** | **Facilities with laboratories** |
| --- | --- | --- |
|  | - ABO blood groups and Rhesus (Rh) factor typing - Ketone - Albumin - Bilirubin - Human Chorionic Gonadotropin hormone - ESR | - Histology/cytology – for infection, neoplasm and inflammatory disorder - ABO blood groups and Rhesus (Rh) factor typing - D-dimer, fibrinogen - Coomb’s test (direct and indirect) - RBC Antibody - Kleihauer-Betke acid-elution test - Peripheral blood smear, platelet, total WBC - Sickel cell test - Alanine - Aminotransferase (ALT), Aspartate aminotransferase (AST) - Albumin, globulin, total protein - Bilirubin – total, direct, indirect - Calcium – total, ionized - Creatinine - Phosphate, whole blood lactate - Procalcitonin - Iron studies - Alpha-fetoprotein - TSH - Body fluid microscopy |
| **Disease-specific Tests** | **PERFORM THROUGH RDT and POCT METHOD**  **Noncommunicable diseases:**   - **Diabetes mellitus:** HemoglobulinA1C   **Communicable diseases**   - ***Chagas disease****: Trypanosoma cruzi* IgG antibody - **Cholera:** Vibrio cholera antigen - **COVID-19**: SARS-CoV-2 antigen - **Hepatitis B virus infection**: HBsAg, HBeAg - **Hepatitis C virus infection**: anti-HCV - **Hepatitis E virus infection:** anti-HEV IgM - **HIV** - anti‑HIV Ab, anti‑HIV/p24 Ag, nucleic acid test (NAT), CD4 cell enumeration, Cryptococcal antigen, Lipoarabinomannan (LAM) antigen - **Influenza:** Influenza A and B antigen and NAT - **Malaria:** *Plasmodium* spp. antigens - **Streptococcal Pharyngitis:** Group A *Streptococcus* antigen - **Sexually transmitted disease:** Antibodies to *Treponema pallidum* - **TB:** Skin test for TB infection - **Visceral Leishmaniasis**: Recombinant K39 (rK39) antigen | **RECOMMENDED TO PERFORM THROUGH IMMUNOASSAY AND ADVANCE METHOD**  **Noncommunicable diseases:**   - **Cancer:** HCC Screening - Alpha-feto protein, Basic panel of immunohistochemistry for lymphoma and solid organ tumours, BCR-ABL1and ABL1 transcripts, Epidermal growth factor receptor (EGFR) gene mutation, Basic flow cytometry panel of antibodies for leukaemia, Fecal immunochemical test, Human chorionic gonadotrophin (hCG), Lactate dehydrogenase (LDH), Estrogen (ER) and progesterone (6) receptors, Papanicolaou (Pap) smear test, Prostate-specific antigen (PSA), Tyrosine protein kinase receptor (erbB‑2) or human epidermal growth factor receptor 2 (HER-2) overexpression - **Cardiovascular diseases:** Troponin I/T, Lipid profile - **Diabetes mellitus:** HemoglobulinA1C - **Endocrine disorder**: Cortisol (total), Estradiol, Follicle stimulating hormone (FSH), Luteinizing hormone (LH), Parathyroid hormone (PTH), Thyroid stimulating hormone (TSH), Progesterone, Prolactin, 17-Hydroxyprogesterone W(17-OHP)   **Communicable diseases:**   - **Aspergillosi**s: Aspergillus IgG and Aspergillus antigen - **COVID-19**: SARS-CoV – 2 NAT - **Hepatitis B virus infection**: HBsAb, HBsAg, HBeAg, HBV NAT, IgM anti-HBc - **Hepatitis C virus infection**: anti-HCV, HCVcantigen, HCV NAT - **Hepatitis E virus infection**: HEV IgM, HEV NAT - **HIV:** Anti-HIV/p24 Ag, HIV NAT, CD4 Enumeration, Cryptococcal antigen, Histoplasma capsulatum antigen, Lipoarabinomannan (LAM) antigen. - **Human papilloma virus infection:** HPV NAT - **Influenza:** Influenza NAT - **Malaria:** Plasmodium antigen, microscopy - **Neglected tropical diseases**: dengue NS1, dengue IgM, dengue NAT, Kato-Katz Fecal smear, *Trypanosoma cruzi* IgG antibody, Visceral leishmaniasis direct agglutination test, *Pneumocystis jirovecii* nucleic acid test - **Sexually transmitted disease**: Chlamydia trachomatis, Neisseria gonorrhoea NAT, RPR, VDRL, TPHA - **Streptococcal Pharyngitis**: Group A streptococcal Antigen - **Tuberculosis**: light microscopy, fluorescent microscopy, MTB Culture, MTB-NAT, IGRA - **Vaccine preventable disease**: Rubella IgM/IgG, NAT, Measles IgM/IgG, NAT - **Zika virus infection:** zika virus IgM, Zika NAT |

Supplementary table: List of EDL Recommended tests not in EPHS
